# Supplementary material for: Modeling glioblastoma heterogeneity as a dynamic network of cell states
Source: Mol Syst Biol. 2021 Sep 16;17(9):e10105. doi: 10.15252/msb.202010105 (PMC8444284; doi:10.15252/msb.202010105)
Supplement: Supplementary file 6 — Source Data for Figure 5 [file MSB-17-e10105-s004.zip › Figure5A_sourcedata/GSEA_3071/nosigdb_stateV.GseaPreranked.1622795159954/FELDMAN_2020_S-G2.html]

Details for gene set FELDMAN\_2020\_S-G2[GSEA]

|  || Dataset | state43071 |
| Phenotype | NoPhenotypeAvailable |
| Upregulated in class | na\_pos |
| GeneSet | FELDMAN\_2020\_S-G2 |
| Enrichment Score (ES) | 0.6310156 |
| Normalized Enrichment Score (NES) | 4.0429134 |
| Nominal p-value | 0.0 |
| FDR q-value | 0.0 |
| FWER p-Value | 0.0 |
Table: GSEA Results Summary

  

Fig 1: Enrichment plot: FELDMAN\_2020\_S-G2      
 Profile of the Running ES Score & Positions of GeneSet Members on the Rank Ordered List

  

| PROBE | GENE SYMBOL | GENE\_TITLE | RANK IN GENE LIST | RANK METRIC SCORE | RUNNING ES | CORE ENRICHMENT || 1 | HIST1H1B |  |  | 0 | 2.500 | 0.0099 | Yes |
| 2 | HIST1H4C |  |  | 1 | 2.442 | 0.0195 | Yes |
| 3 | ATAD2 |  |  | 2 | 2.214 | 0.0283 | Yes |
| 4 | ESCO2 |  |  | 3 | 2.193 | 0.0369 | Yes |
| 5 | HMGB2 |  |  | 5 | 2.011 | 0.0447 | Yes |
| 6 | NUSAP1 |  |  | 6 | 2.001 | 0.0526 | Yes |
| 7 | HIST1H1C |  |  | 7 | 1.976 | 0.0604 | Yes |
| 8 | PCNA |  |  | 10 | 1.908 | 0.0675 | Yes |
| 9 | PTPRZ1 |  |  | 13 | 1.865 | 0.0745 | Yes |
| 10 | TOP2A |  |  | 14 | 1.842 | 0.0818 | Yes |
| 11 | HIST1H1E |  |  | 15 | 1.830 | 0.0890 | Yes |
| 12 | DHFR |  |  | 18 | 1.795 | 0.0957 | Yes |
| 13 | HIST1H1D |  |  | 19 | 1.786 | 0.1028 | Yes |
| 14 | NCAPG |  |  | 21 | 1.760 | 0.1095 | Yes |
| 15 | RAD51AP1 |  |  | 22 | 1.757 | 0.1165 | Yes |
| 16 | SYNE2 |  |  | 25 | 1.732 | 0.1229 | Yes |
| 17 | H2AFX |  |  | 26 | 1.725 | 0.1297 | Yes |
| 18 | CENPF |  |  | 28 | 1.705 | 0.1363 | Yes |
| 19 | TYMS |  |  | 30 | 1.683 | 0.1427 | Yes |
| 20 | HIST2H2AC |  |  | 31 | 1.681 | 0.1494 | Yes |
| 21 | RRM2 |  |  | 34 | 1.660 | 0.1555 | Yes |
| 22 | MYBL2 |  |  | 35 | 1.651 | 0.1620 | Yes |
| 23 | CENPU |  |  | 36 | 1.645 | 0.1685 | Yes |
| 24 | MKI67 |  |  | 45 | 1.598 | 0.1733 | Yes |
| 25 | HELLS |  |  | 48 | 1.588 | 0.1791 | Yes |
| 26 | MCM3 |  |  | 49 | 1.586 | 0.1854 | Yes |
| 27 | PBK |  |  | 54 | 1.536 | 0.1907 | Yes |
| 28 | GINS2 |  |  | 57 | 1.528 | 0.1963 | Yes |
| 29 | MXD3 |  |  | 62 | 1.500 | 0.2014 | Yes |
| 30 | GTSE1 |  |  | 66 | 1.491 | 0.2067 | Yes |
| 31 | CDK1 |  |  | 69 | 1.488 | 0.2122 | Yes |
| 32 | SMC2 |  |  | 73 | 1.466 | 0.2174 | Yes |
| 33 | SPC25 |  |  | 76 | 1.442 | 0.2227 | Yes |
| 34 | IGFBP3 |  |  | 78 | 1.437 | 0.2282 | Yes |
| 35 | UBE2T |  |  | 79 | 1.432 | 0.2338 | Yes |
| 36 | HIST1H2AH |  |  | 80 | 1.424 | 0.2395 | Yes |
| 37 | FBXO5 |  |  | 82 | 1.422 | 0.2449 | Yes |
| 38 | CLSPN |  |  | 84 | 1.417 | 0.2503 | Yes |
| 39 | SCRG1 |  |  | 87 | 1.404 | 0.2554 | Yes |
| 40 | MCM7 |  |  | 88 | 1.400 | 0.2610 | Yes |
| 41 | DEK |  |  | 94 | 1.384 | 0.2654 | Yes |
| 42 | CDCA2 |  |  | 96 | 1.382 | 0.2707 | Yes |
| 43 | FABP7 |  |  | 98 | 1.372 | 0.2759 | Yes |
| 44 | RRM1 |  |  | 99 | 1.364 | 0.2813 | Yes |
| 45 | CARHSP1 |  |  | 101 | 1.362 | 0.2865 | Yes |
| 46 | KIFC1 |  |  | 113 | 1.338 | 0.2896 | Yes |
| 47 | CENPM |  |  | 114 | 1.337 | 0.2949 | Yes |
| 48 | TACC3 |  |  | 116 | 1.334 | 0.2999 | Yes |
| 49 | GPM6B |  |  | 121 | 1.326 | 0.3044 | Yes |
| 50 | SMC4 |  |  | 127 | 1.320 | 0.3086 | Yes |
| 51 | FEN1 |  |  | 139 | 1.296 | 0.3115 | Yes |
| 52 | RTKN2 |  |  | 143 | 1.285 | 0.3160 | Yes |
| 53 | GMNN |  |  | 146 | 1.280 | 0.3207 | Yes |
| 54 | MIS18BP1 |  |  | 159 | 1.247 | 0.3232 | Yes |
| 55 | TMPO |  |  | 161 | 1.243 | 0.3279 | Yes |
| 56 | CENPK |  |  | 162 | 1.241 | 0.3328 | Yes |
| 57 | LMNB1 |  |  | 165 | 1.238 | 0.3373 | Yes |
| 58 | DNMT1 |  |  | 166 | 1.237 | 0.3422 | Yes |
| 59 | KIF23 |  |  | 167 | 1.235 | 0.3471 | Yes |
| 60 | CDCA3 |  |  | 169 | 1.230 | 0.3517 | Yes |
| 61 | RFC4 |  |  | 171 | 1.227 | 0.3564 | Yes |
| 62 | NASP |  |  | 180 | 1.212 | 0.3596 | Yes |
| 63 | GGH |  |  | 182 | 1.207 | 0.3641 | Yes |
| 64 | DBF4 |  |  | 189 | 1.197 | 0.3677 | Yes |
| 65 | CDKN2D |  |  | 190 | 1.196 | 0.3724 | Yes |
| 66 | KIF4A |  |  | 191 | 1.193 | 0.3771 | Yes |
| 67 | NUF2 |  |  | 192 | 1.189 | 0.3818 | Yes |
| 68 | PXMP2 |  |  | 195 | 1.180 | 0.3861 | Yes |
| 69 | USP1 |  |  | 202 | 1.168 | 0.3895 | Yes |
| 70 | ZWINT |  |  | 204 | 1.166 | 0.3939 | Yes |
| 71 | RNASEH2B |  |  | 205 | 1.166 | 0.3985 | Yes |
| 72 | ASPM |  |  | 207 | 1.163 | 0.4029 | Yes |
| 73 | RBBP8 |  |  | 210 | 1.162 | 0.4071 | Yes |
| 74 | PRC1 |  |  | 211 | 1.161 | 0.4117 | Yes |
| 75 | KIF22 |  |  | 213 | 1.157 | 0.4160 | Yes |
| 76 | UBE2C |  |  | 214 | 1.154 | 0.4206 | Yes |
| 77 | BTG3 |  |  | 226 | 1.141 | 0.4229 | Yes |
| 78 | CDC45 |  |  | 228 | 1.138 | 0.4272 | Yes |
| 79 | ASF1B |  |  | 237 | 1.130 | 0.4301 | Yes |
| 80 | NDC80 |  |  | 240 | 1.129 | 0.4342 | Yes |
| 81 | TPX2 |  |  | 248 | 1.122 | 0.4372 | Yes |
| 82 | SMC1A |  |  | 251 | 1.120 | 0.4412 | Yes |
| 83 | SAC3D1 |  |  | 252 | 1.118 | 0.4456 | Yes |
| 84 | CDH2 |  |  | 255 | 1.115 | 0.4496 | Yes |
| 85 | ENC1 |  |  | 265 | 1.097 | 0.4522 | Yes |
| 86 | BRCA2 |  |  | 274 | 1.089 | 0.4549 | Yes |
| 87 | CKAP2L |  |  | 285 | 1.078 | 0.4572 | Yes |
| 88 | BEX1 |  |  | 287 | 1.077 | 0.4612 | Yes |
| 89 | HIRIP3 |  |  | 289 | 1.077 | 0.4653 | Yes |
| 90 | AURKB |  |  | 297 | 1.068 | 0.4681 | Yes |
| 91 | LYPD1 |  |  | 299 | 1.067 | 0.4721 | Yes |
| 92 | ARHGAP11A |  |  | 303 | 1.064 | 0.4757 | Yes |
| 93 | STMN1 |  |  | 307 | 1.062 | 0.4793 | Yes |
| 94 | CCNA2 |  |  | 309 | 1.057 | 0.4833 | Yes |
| 95 | CKAP2 |  |  | 312 | 1.053 | 0.4871 | Yes |
| 96 | PSIP1 |  |  | 313 | 1.053 | 0.4912 | Yes |
| 97 | DUT |  |  | 314 | 1.050 | 0.4954 | Yes |
| 98 | TMEM106C |  |  | 334 | 1.034 | 0.4957 | Yes |
| 99 | RFC2 |  |  | 337 | 1.025 | 0.4993 | Yes |
| 100 | S100B |  |  | 361 | 0.996 | 0.4987 | Yes |
| 101 | CDKN1A |  |  | 363 | 0.993 | 0.5024 | Yes |
| 102 | DNAJC9 |  |  | 373 | 0.979 | 0.5045 | Yes |
| 103 | PARP1 |  |  | 374 | 0.978 | 0.5083 | Yes |
| 104 | DSN1 |  |  | 377 | 0.975 | 0.5118 | Yes |
| 105 | PSMC3IP |  |  | 382 | 0.968 | 0.5148 | Yes |
| 106 | RPA3 |  |  | 395 | 0.953 | 0.5162 | Yes |
| 107 | HJURP |  |  | 406 | 0.945 | 0.5179 | Yes |
| 108 | NUDT1 |  |  | 417 | 0.936 | 0.5196 | Yes |
| 109 | MAD2L1 |  |  | 421 | 0.932 | 0.5227 | Yes |
| 110 | KPNA2 |  |  | 435 | 0.919 | 0.5238 | Yes |
| 111 | CENPE |  |  | 437 | 0.918 | 0.5272 | Yes |
| 112 | VRK1 |  |  | 445 | 0.911 | 0.5294 | Yes |
| 113 | CENPH |  |  | 448 | 0.908 | 0.5326 | Yes |
| 114 | ORC6 |  |  | 449 | 0.908 | 0.5362 | Yes |
| 115 | CLU |  |  | 464 | 0.900 | 0.5369 | Yes |
| 116 | SHCBP1 |  |  | 465 | 0.900 | 0.5405 | Yes |
| 117 | HMGN2 |  |  | 466 | 0.900 | 0.5441 | Yes |
| 118 | PCSK1N |  |  | 476 | 0.887 | 0.5458 | Yes |
| 119 | MND1 |  |  | 480 | 0.885 | 0.5487 | Yes |
| 120 | SCD5 |  |  | 485 | 0.879 | 0.5513 | Yes |
| 121 | AKAP12 |  |  | 487 | 0.878 | 0.5546 | Yes |
| 122 | UBE2S |  |  | 514 | 0.854 | 0.5528 | Yes |
| 123 | DCK |  |  | 516 | 0.854 | 0.5560 | Yes |
| 124 | RFC5 |  |  | 527 | 0.849 | 0.5573 | Yes |
| 125 | BIRC5 |  |  | 541 | 0.842 | 0.5581 | Yes |
| 126 | TCF19 |  |  | 542 | 0.842 | 0.5614 | Yes |
| 127 | PRKDC |  |  | 554 | 0.828 | 0.5625 | Yes |
| 128 | GPM6A |  |  | 575 | 0.815 | 0.5617 | Yes |
| 129 | CMC2 |  |  | 580 | 0.812 | 0.5641 | Yes |
| 130 | HMGB1 |  |  | 583 | 0.810 | 0.5669 | Yes |
| 131 | DIAPH3 |  |  | 585 | 0.808 | 0.5699 | Yes |
| 132 | TK1 |  |  | 591 | 0.806 | 0.5721 | Yes |
| 133 | TRA2B |  |  | 596 | 0.802 | 0.5745 | Yes |
| 134 | ACYP1 |  |  | 609 | 0.794 | 0.5752 | Yes |
| 135 | TTYH1 |  |  | 611 | 0.794 | 0.5782 | Yes |
| 136 | CDCA4 |  |  | 615 | 0.790 | 0.5807 | Yes |
| 137 | CENPA |  |  | 627 | 0.786 | 0.5816 | Yes |
| 138 | SIVA1 |  |  | 646 | 0.774 | 0.5811 | Yes |
| 139 | PLAT |  |  | 651 | 0.773 | 0.5833 | Yes |
| 140 | HNRNPD |  |  | 659 | 0.769 | 0.5850 | Yes |
| 141 | SKA3 |  |  | 668 | 0.765 | 0.5864 | Yes |
| 142 | WDR34 |  |  | 671 | 0.762 | 0.5890 | Yes |
| 143 | ARL6IP6 |  |  | 702 | 0.745 | 0.5860 | Yes |
| 144 | SMC3 |  |  | 708 | 0.743 | 0.5879 | Yes |
| 145 | TFDP1 |  |  | 711 | 0.742 | 0.5905 | Yes |
| 146 | NETO2 |  |  | 717 | 0.740 | 0.5924 | Yes |
| 147 | ATAD5 |  |  | 721 | 0.739 | 0.5947 | Yes |
| 148 | PGP |  |  | 727 | 0.732 | 0.5966 | Yes |
| 149 | TEX30 |  |  | 730 | 0.730 | 0.5991 | Yes |
| 150 | HAT1 |  |  | 741 | 0.727 | 0.6000 | Yes |
| 151 | PIN1 |  |  | 746 | 0.725 | 0.6021 | Yes |
| 152 | TUBG1 |  |  | 776 | 0.712 | 0.5991 | Yes |
| 153 | CEP78 |  |  | 791 | 0.704 | 0.5991 | Yes |
| 154 | SNRNP25 |  |  | 795 | 0.702 | 0.6013 | Yes |
| 155 | DTYMK |  |  | 800 | 0.700 | 0.6032 | Yes |
| 156 | TRIP13 |  |  | 804 | 0.698 | 0.6054 | Yes |
| 157 | TUBB |  |  | 836 | 0.685 | 0.6019 | Yes |
| 158 | H2AFZ |  |  | 851 | 0.677 | 0.6018 | Yes |
| 159 | RNASEH2A |  |  | 859 | 0.674 | 0.6031 | Yes |
| 160 | PARP2 |  |  | 864 | 0.673 | 0.6050 | Yes |
| 161 | C19orf48 |  |  | 893 | 0.661 | 0.6020 | Yes |
| 162 | SOX9 |  |  | 905 | 0.658 | 0.6024 | Yes |
| 163 | MELK |  |  | 915 | 0.653 | 0.6032 | Yes |
| 164 | MZT1 |  |  | 917 | 0.652 | 0.6056 | Yes |
| 165 | SRSF7 |  |  | 921 | 0.650 | 0.6075 | Yes |
| 166 | UBE2I |  |  | 932 | 0.647 | 0.6081 | Yes |
| 167 | H2AFV |  |  | 933 | 0.647 | 0.6107 | Yes |
| 168 | KIF20B |  |  | 934 | 0.647 | 0.6132 | Yes |
| 169 | PTTG1 |  |  | 936 | 0.647 | 0.6156 | Yes |
| 170 | TMEM237 |  |  | 952 | 0.639 | 0.6151 | Yes |
| 171 | CENPN |  |  | 953 | 0.638 | 0.6176 | Yes |
| 172 | ZFAND6 |  |  | 958 | 0.638 | 0.6193 | Yes |
| 173 | CKS2 |  |  | 966 | 0.634 | 0.6205 | Yes |
| 174 | PRCP |  |  | 983 | 0.626 | 0.6197 | Yes |
| 175 | HAUS1 |  |  | 987 | 0.624 | 0.6216 | Yes |
| 176 | LRR1 |  |  | 991 | 0.623 | 0.6235 | Yes |
| 177 | RANBP1 |  |  | 999 | 0.620 | 0.6245 | Yes |
| 178 | LSM4 |  |  | 1003 | 0.619 | 0.6264 | Yes |
| 179 | TUBA1B |  |  | 1012 | 0.617 | 0.6272 | Yes |
| 180 | MIS18A |  |  | 1023 | 0.614 | 0.6277 | Yes |
| 181 | RNPS1 |  |  | 1035 | 0.612 | 0.6279 | Yes |
| 182 | ZWILCH |  |  | 1048 | 0.609 | 0.6279 | Yes |
| 183 | MAP3K7CL |  |  | 1055 | 0.606 | 0.6291 | Yes |
| 184 | TMEM178A |  |  | 1073 | 0.601 | 0.6281 | Yes |
| 185 | DONSON |  |  | 1082 | 0.597 | 0.6289 | Yes |
| 186 | SRSF2 |  |  | 1084 | 0.597 | 0.6310 | Yes |
| 187 | DEPDC1 |  |  | 1179 | 0.569 | 0.6145 | No |
| 188 | ANP32B |  |  | 1187 | 0.566 | 0.6154 | No |
| 189 | HIST1H2AM |  |  | 1196 | 0.563 | 0.6160 | No |
| 190 | HNRNPF |  |  | 1216 | 0.559 | 0.6144 | No |
| 191 | EXOSC8 |  |  | 1230 | 0.553 | 0.6140 | No |
| 192 | ITM2B |  |  | 1244 | 0.547 | 0.6136 | No |
| 193 | RMI2 |  |  | 1261 | 0.542 | 0.6126 | No |
| 194 | TM7SF2 |  |  | 1273 | 0.539 | 0.6125 | No |
| 195 | CMSS1 |  |  | 1350 | 0.520 | 0.5994 | No |
| 196 | RAD23A |  |  | 1356 | 0.518 | 0.6005 | No |
| 197 | SPDL1 |  |  | 1397 | 0.507 | 0.5945 | No |
| 198 | TUBA1A |  |  | 1403 | 0.504 | 0.5955 | No |
| 199 | YWHAH |  |  | 1407 | 0.502 | 0.5969 | No |
| 200 | GGCT |  |  | 1414 | 0.501 | 0.5977 | No |
| 201 | POLR3K |  |  | 1476 | 0.487 | 0.5875 | No |
| 202 | CKLF |  |  | 1480 | 0.486 | 0.5888 | No |
| 203 | SUPT16H |  |  | 1500 | 0.480 | 0.5869 | No |
| 204 | CKS1B |  |  | 1502 | 0.479 | 0.5886 | No |
| 205 | CEP55 |  |  | 1508 | 0.479 | 0.5895 | No |
| 206 | HNRNPAB |  |  | 1541 | 0.471 | 0.5850 | No |
| 207 | POP7 |  |  | 1591 | 0.461 | 0.5770 | No |
| 208 | SSRP1 |  |  | 1634 | 0.451 | 0.5705 | No |
| 209 | TUBB4B |  |  | 1659 | 0.444 | 0.5674 | No |
| 210 | IER2 |  |  | 1742 | 0.428 | 0.5528 | No |
| 211 | DERA |  |  | 1749 | 0.428 | 0.5533 | No |
| 212 | DCTPP1 |  |  | 1796 | 0.418 | 0.5458 | No |
| 213 | SNRPG |  |  | 1825 | 0.414 | 0.5418 | No |
| 214 | PSAP |  |  | 1855 | 0.410 | 0.5377 | No |
| 215 | PLAU |  |  | 1863 | 0.408 | 0.5379 | No |
| 216 | CENPW |  |  | 1959 | 0.395 | 0.5205 | No |
| 217 | MALAT1 |  |  | 1988 | 0.391 | 0.5165 | No |
| 218 | PA2G4 |  |  | 2112 | 0.372 | 0.4935 | No |
| 219 | ANLN |  |  | 2125 | 0.371 | 0.4926 | No |
| 220 | RHEB |  |  | 2192 | 0.363 | 0.4809 | No |
| 221 | HNRNPDL |  |  | 2203 | 0.362 | 0.4803 | No |
| 222 | TMEM158 |  |  | 2340 | 0.343 | 0.4546 | No |
| 223 | PAICS |  |  | 2403 | 0.334 | 0.4435 | No |
| 224 | HPRT1 |  |  | 2404 | 0.334 | 0.4449 | No |
| 225 | CHCHD3 |  |  | 2434 | 0.330 | 0.4404 | No |
| 226 | SRSF3 |  |  | 2732 | 0.297 | 0.3824 | No |
| 227 | HMGN1 |  |  | 2742 | 0.295 | 0.3818 | No |
| 228 | SLC25A5 |  |  | 2796 | 0.290 | 0.3724 | No |
| 229 | BUB3 |  |  | 2806 | 0.288 | 0.3717 | No |
| 230 | SAE1 |  |  | 2872 | 0.283 | 0.3599 | No |
| 231 | HSPB11 |  |  | 3163 | 0.254 | 0.3032 | No |
| 232 | ATP6V1G1 |  |  | 3316 | -0.270 | 0.2740 | No |
| 233 | EIF5 |  |  | 3375 | -0.278 | 0.2635 | No |
| 234 | COMMD6 |  |  | 3394 | -0.280 | 0.2610 | No |
| 235 | PFDN5 |  |  | 3451 | -0.290 | 0.2510 | No |
| 236 | CNPY2 |  |  | 3603 | -0.316 | 0.2222 | No |
| 237 | EEF1A1 |  |  | 3617 | -0.318 | 0.2209 | No |
| 238 | RABAC1 |  |  | 3622 | -0.318 | 0.2213 | No |
| 239 | PRELID1 |  |  | 3709 | -0.332 | 0.2055 | No |
| 240 | BAMBI |  |  | 3718 | -0.334 | 0.2053 | No |
| 241 | YPEL5 |  |  | 3745 | -0.339 | 0.2014 | No |
| 242 | ARL6IP5 |  |  | 3781 | -0.346 | 0.1958 | No |
| 243 | EIF2S1 |  |  | 3809 | -0.351 | 0.1918 | No |
| 244 | SDCBP |  |  | 3840 | -0.357 | 0.1873 | No |
| 245 | C4orf3 |  |  | 3884 | -0.364 | 0.1801 | No |
| 246 | TUBB6 |  |  | 3904 | -0.368 | 0.1778 | No |
| 247 | EEF2 |  |  | 3913 | -0.369 | 0.1777 | No |
| 248 | RPS27L |  |  | 3927 | -0.373 | 0.1766 | No |
| 249 | CMTM7 |  |  | 4056 | -0.400 | 0.1526 | No |
| 250 | DDIT4 |  |  | 4182 | -0.432 | 0.1295 | No |
| 251 | PMAIP1 |  |  | 4312 | -0.476 | 0.1057 | No |
| 252 | PLP2 |  |  | 4366 | -0.490 | 0.0970 | No |
| 253 | IFITM3 |  |  | 4424 | -0.509 | 0.0877 | No |
| 254 | FHL2 |  |  | 4451 | -0.521 | 0.0846 | No |
| 255 | RRS1 |  |  | 4502 | -0.542 | 0.0768 | No |
| 256 | PSMG1 |  |  | 4573 | -0.574 | 0.0651 | No |
| 257 | PFDN2 |  |  | 4585 | -0.579 | 0.0652 | No |
| 258 | RPL34 |  |  | 4660 | -0.624 | 0.0529 | No |
| 259 | POLD4 |  |  | 4705 | -0.646 | 0.0467 | No |
| 260 | RAB13 |  |  | 4786 | -0.701 | 0.0336 | No |
| 261 | SMS |  |  | 4842 | -0.750 | 0.0256 | No |
| 262 | MAGED2 |  |  | 4884 | -0.793 | 0.0205 | No |
| 263 | KLF6 |  |  | 4933 | -0.849 | 0.0143 | No |
| 264 | C12orf75 |  |  | 4940 | -0.858 | 0.0165 | No |
| 265 | HMGA1 |  |  | 4962 | -0.878 | 0.0158 | No |
| 266 | IGFBP5 |  |  | 4978 | -0.897 | 0.0164 | No |
| 267 | CTGF |  |  | 5070 | -1.073 | 0.0025 | No |
| 268 | FTL |  |  | 5072 | -1.077 | 0.0065 | No |
| 269 | PRDX1 |  |  | 5104 | -1.163 | 0.0050 | No |
| 270 | SERPINF1 |  |  | 5158 | -1.351 | -0.0002 | No |
| 271 | PHLDA2 |  |  | 5163 | -1.384 | 0.0044 | No |
| 272 | IL11 |  |  | 5170 | -1.414 | 0.0088 | No |
| 273 | MIAT |  |  | 5225 | -1.704 | 0.0048 | No |
| 274 | RGCC |  |  | 5264 | -2.264 | 0.0062 | No |
Table: GSEA details [plain text format]

  

Fig 2: FELDMAN\_2020\_S-G2: Random ES distribution      
 Gene set null distribution of ES for **FELDMAN\_2020\_S-G2**
